# Exosomes derived from mesenchymal stem cells overexpressing miR-210 inhibits neuronal inflammation and contribute to neurite outgrowth through modulating microglia polarization

**DOI:** 10.1515/med-2022-0618

**Published:** 2023-01-04

**Authors:** Qing-hua Xiong, Lei Zhao, Guan-qun Wan, Yun-gang Hu, Xiao-lin Li

**Affiliations:** Department of Plastic and Maxillofacial Surgery, Jiangxi People’s Hospital/Jiangxi Province Key Laboratory of Maxillofacial Plastic and Reconstruction, Nanchang, China

**Keywords:** exosomes, mesenchymal stem cells, microglia polarization, miR-210

## Abstract

Inflammatory responses play a critical role in the progress of neurodegenerative disorders. MSC-Exos is considered to have an anti-inflammatory effect on the treatment strategy for brain injury. However, the therapeutic effect and possible mechanism of Exosomal miR-210 on microglia polarization-induced neuroinflammation and neurite outgrowth have not been reported. MSC-Exos were isolated by ultracentrifugation, identified by Nanosight NS300, transmission electron microscopy, and western bolt. *In vitro*, to explore the protective mechanism of MSC-Exos against neuroinflammation, the microglial BV2 cell was exposed to lipopolysaccharide to assess inflammatory changes. The intake of 1,1’-dioctadecyl-3,3,3’,3’-tetramethylindocarbocyanine perchlorate (Dil)-MSC-Exos into microglia was observed by fluorescence microscopy. The results showed that Exosomal miR-210 treatment significantly inhibited the production of nitric oxide and pro-inflammatory cytokines. Exosomal miR-210 treatment also increased the number of M2 microglia cells and inhibited M1 microglia polarization. In addition, western blot demonstrated that Exosomal miR-210 reduced neuronal apoptosis. Thus, Exosomal miR-210 attenuated neuronal inflammation and promoted neurite outgrowth. Exosomal miR-210 from MSCs attenuated neuronal inflammation and contributed to neurogenesis possibly by inhibiting microglial M1 polarization.

## 1 Introduction

Neuroinflammation is a term commonly used for inflammation of neural tissue, particularly the central nervous system [1]. Emerging evidence indicates that neuroinflammation is a significant pathological process triggering a series of molecular and cellular events following neurodegenerative disorders. Neuroinflammation plays an essential role in secondary damage to the brain [2,3]. In neuroinflammation, microglia mediate a series of immuno-modulating process [4,5] and release inflammatory mediators, such as cytokines and chemokines [6]. Molfino et al. further suggested that neuroinflammation is triggered and long-lasting through the activation of microglia [7].

The activation and regulation of microglia are involved in the pathological development of a variety of central nervous diseases [8–10]. Microglia can be activated to shift the M1/M2 phenotype to trigger different immune modulations [11,12]. M1 phenotype typically secretes pro-inflammatory cytokines and promotes neuroinflammation [13]. The alternative M2 phenotype secretes anti-inflammatory cytokines including TGF-β, IL-10, and IL-4, which are favorable to neurogenesis, while activation of the M2 phenotype promotes the production of anti-inflammatory cytokines such as IL-10 and TGF-β, resulting in anti-neuroinflammation and neurogenesis [14]. Therefore, the modulation of microglia polarization has been suggested as a promising therapeutic approach for neuroinflammation.

Lipopolysaccharide (LPS), an endotoxin, stimulates M1 microglia polarization, while IL-4 or IL-10 modulates the M2 phenotype [15]. M1 inhibitive agents have been used against neurodegenerative diseases and inhibited inflammatory. And the inhibitive agents have few beneficial effects. Therefore, the activation of the M2 phenotype might also play a vital role in improving the beneficial microenvironment in the brain [16].

In a recent study, the transplantation of mesenchymal stem cells (MSCs) has been studied in various neurodegenerative diseases for their immunomodulatory properties. It has been confirmed that paracrine mechanisms are more likely involved in anti-inflammation and immunomodulation. Especially, exosomes (EXOs) may play a vital role in the treatment of several neurodegenerative diseases [17]. MSC-Exos are released from cells with a size of 50–150 nm and pass through the blood–brain barrier freely [18]. MSC-Exos have been considered as essential modulators involved in intercellular communication. EXOs deliver cargos such as RNAs, microRNAs (miRNAs), proteins, and cytokines from the originating cells to the recipient cells, thereby modifying many diseases’ occurrence, progression, and prognosis [19–22]. MSC-Exos deliver their active molecules into microglia through ligand–receptor interaction patterns, direct membrane fusion, endocytosis, or phagocytosis. It has been shown that MSC-Exos have extremely anti-inflammatory and immunosuppressive effects on various neurological diseases through modulating microglia activation [23,24]. Therefore, MSC-Exos have broad possibilities in the treatment of neurodegenerative diseases.

It has been demonstrated that miRNAs might play an essential role in anti-inflammation and immunomodulation by EXOs [25,26]. In recent studies, miR-210 inhibited the inflammatory responses in LPS-induced microglia and regulated the shift of M1/M2 phenotypes.

The function of miR-210 on microglia-induced neuroinflammation has not been fully revealed [27,28]. Therefore, we have focused on EXOs derived from MSCs overexpressing miR-210 that could inhibit neuroinflammation and promote neurogenesis effectively. Furthermore, our study uncovered that Exosomal miR-210 could suppress neuroinflammation and contribute to neurite outgrowth by regulating microglia polarization, meaning Exosomal miR-210 may play an essential role in neuroinflammation.

## 2 Methods

### 2.1 Cell culture

MSCs were isolated and cultured in a culture medium. The murine microglial BV2 cell line was purchased from the China Center for Type Culture Collection. The mouse hippocampal neuron cell line HT22 was obtained from the Shanghai iCell Biotechnology Company. The cell culture media are described below: DMEM (for BV2 cells, Invitrogen, USA) or DMEM/F12 (for MSCs, Invitrogen, USA), 10% EXO-depleted fetal bovine serum (Invitrogen, USA), 100 U/mL penicillin–streptomycin solutions (Thermo Fisher Scientific, USA). Cells were seeded in a 25 cm^2^ cell culture flask at 37°C, 5% CO_2_. Then the medium was changed every 2 days. When cells reached 80% confluence, they were digested by 0.25% trypsin–ethylenediaminetetraacetic acid (EDTA) digestion (Sigma-Aldrich, USA). The passage 3–4 cells were used for further experiments.

### 2.2 Isolation and identification of bone mesenchymal stem cells EXO

MSCs cell culture medium was collected every 48 h. First, the collected culture medium was centrifuged at 300×*g* for 10 min to eliminate the cell pellets at 4°C. Then, the supernatant was centrifuged at 2,000×*g* for 10 min to further remove the cell debris. Next, the supernatant was again centrifuged at 10,000×*g* for 30 min. Finally, the cell supernatant was filtered through a 0.22 μm filter (Merck Millipore, Germany) to remove the cell debris.

Next, the supernatant was collected and transferred to new tubes (Beckman, USA). Then the supernatant was ultracentrifuged at 120,000×*g* in an SW70Ti rotor (Beckman, Pasadena, CA) for 140 min. Next, the phosphate-buffered saline (PBS) resuspended the EXO-enriched pellet and was ultracentrifuged again. Finally, 200 μL cold PBS buffer was used to resuspend the EXOs.

The BCA protein assay kit (Thermo Fisher Scientific, USA) was used to determine the protein content of EXOs. The solution was stored at −80°C.

To analyze the particle size of EXOs, MSC-Exos was detected by NanoSight NS300 (Malvern Instruments, UK). The transmission electron microscopy (TEM) (FEI Tecnai 12, Philips, USA) identified the obtained EXOs. Exosomal surface markers such as CD63, TSG101, and cytochrome C were identified by western blotting.

### 2.3 miRNA mimic transfection

The resuspended MSCs EXOs were diluted in Gene Pulser^®^ electroporation buffer (Bio-Rad) at a ratio of 1:1. A final amount of 150 pmol of miR-210 mimic or NC mimic (GenePharma) was added to 0.5 μg/mL MSCs EXO sample. The mixture was transferred to a cold 0.2 cm electroporation cuvette and incubated at 100 μF at 0.150 kV. EXOs were treated with one unit of RNase H to eliminate free floating miR-210 mimics outside the EXOs and were re-isolated using Exoquick TC™.

### 2.4 EXOs uptake assay

Following the manufacturer’s instructions, the MSCs-Exo was labeled with the PKH26 Fluorescent (Thermo Fisher Scientific, USA) (1 µL 1,1’-dioctadecyl-3,3,3’,3’-tetramethylindocarbocyanine perchlorate (DIL) for 100 µL solution). MSCs-Exo was incubated at room temperature for 20 min. Next, the supernatant was abandoned, and the new cold PBS was used to resuspend PKH26-labeled EXOs. According to the protocol, the EXO sample was ultracentrifuged at 100,000×*g* for 70 min to wash the dye. PKH26-MSCs-Exo/NC mimic or PKH26-MSCs-Exo/miR-210 mimic was co-cultured with microglia for 24 h, and the location of PKH26-MSCs-Exo was observed by fluorescence microscopy (Carl Zeiss, Germany).

### 2.5 Immunofluorescence staining

The 4% paraformaldehyde fixed cells for 10 min at room temperature. Next, cells were blocked with 5% bovine serum albumin (BSA) for 1 h at room temperature. After that, the antibody of Ym1/2 (ab192029, 1:100; Abcam, UK) and Cox2 (ab179800, 1:100; Abcam, UK) or β-tubulin III (ab18207, 1:2,000: Abcam, UK) was used to incubate the cells for 12 h at 4°C. Next, the cells were washed three times with PBS. Next, the Cy3-conjugated AffiniPure IgG (HL) secondary antibody (1:800, Abcam, UK) was used to treat cells for 1 h in the dark and was washed three times with PBS. Next, the sections were stained by 4′,6-diamidino-2-phenylindole (DAPI) for 5 min and were detected using an Afv10i confocal microscope (Keyence, Japan) at 495, 565, and 400 nm. The expressions of COX2 and Ym1/2, the axon length, and neurite branches of neuronal cells were quantitatively analyzed using ImageJ (ImageJ 1.48v, NIH).

### 2.6 Flow cytometry

After LPS treatment, neuronal cells were cultured with Exo/NC mimic or Exo/miR-210 mimic. Then, the cells were suspended using trypsin–EDTA and centrifuged at 1,000 rpm for 5 min. Next, the cells were washed using cooled PBS. Thereafter, the cells were stained with fluorescein isothiocyanate-conjugated annexin-V and phycoerythrin-conjugated propidium iodide from the Annexin-V staining kit (BD Pharmingen™, USA) [29]. After incubation, the samples were detected by BD FACSuite (BD Life Sciences, USA).

### 2.7 Western blotting

The protein extraction kit (KeyGEN, China) extracted proteins from the EXOs or cells. Then, a BCA assay kit (Thermo Fisher Scientific, USA) detected the concentrations of proteins. The antibody of β-actin (#3700, 1:5,000, CST, USA) was used as a loading control. About 15 μg of protein was separated by 10% SDS-PAGE and transferred to PVDF membranes (EpiZyme, China). Next, the membranes blocked the non-specific antigen in 5% BSA for 1 h at room temperature. After that, the membranes were incubated with specific antibodies at 4°C for 12 h. Then, the membranes were washed with tris buffered saline with Tween-20 three times, and followed by incubation with secondary antibodies (1:5,000, Abcam, USA) for 2 h. After washing, the bands were detected using Gel-Pro Analyzer software (Media Cybernetics, USA).

### 2.8 Quantitative real-time PCR

According to the manufacturer’s instructions, total RNA was extracted from the cells or EXOs using RNAiso (Takara, Otsu, Japan). SYBR-Green qPCR Mix (Nanjing, China) was used for qPCR mRNA quantification. The abundance of miR-210, TNF-α, IL-1β, iNOS, IL-4, CD206, Arg1, TGF-β normalized to U6 small nuclear RNA or GAPDH. Moreover, we analyzed the data using the formula of 2^−ΔΔCt^. Appropriate primers are listed in Table A1.

### 2.9 CCK-8 assay

The 96-well cell culture plate was used in the CCK-8 assay. First, microglia were plated into a cell culture plate with 2,500 cells per well. Next, the CCK-8 solution (Sigma-Aldrich, USA) was added to the plate and incubated at 37°C for 1 h. After that, cells were measured at 450 nm OD values using a Microplate Reader (Bio-Rad, USA).

### 2.10 Analysis of total nitric oxide (NO) concentration

As we all know, the stable products of NO metabolism are nitrite ( {\text{NO}}_{2}^{-}] ) and nitrate ( {\text{NO}}_{3}^{-}] ) [30]. Therefore, {\text{NO}}_{2}^{-}] and {\text{NO}}_{3}^{-}] are used to detect the concentration of NO. The supernatant of LPS-stimulated microglia was collected, and the nitrite concentration was measured according to Griess (Sigma-Aldrich, Germany) reaction. NO concentrations were detected by spectrophotometric analysis at 540 nm (Biogenet, Austria).

### 2.11 Enzyme-linked immunosorbent assay (ELISA)

Biomarkers were quantified using commercial ELISA kits following the manufacturer’s instructions (Sino Biological Inc., China), and a four-parameter logistic curve was used to fit the standard curve. ELISA kits were used to determine the different IL-6, IL-10, IL-1, and TNF-α levels. A well for samples to be determined, a standard well, and a blank well were set. No enzyme-labeled reagent or sample was added, and 100 µL of samples and 100 µL of standards were added into the well for samples to be determined and standard well, respectively, and mixed well. The supernatant was incubated at 37°C for 1 h. Next, the liquid was discarded, the plate was patted dry, and 100 µL each of fluid A and fluid B was added. Within the last 5 min of the reaction, the OD value of each well was measured sequentially under a standard enzyme instrument (MB-530, China) at a wavelength of 450 nm.

### 2.12 Statistical analysis

We expressed the data as mean ± SD. The results were performed using GraphPad Prism (GraphPad Software, USA). ANOVA was used for comparisons among multiple groups and unpaired *t*-test was used for comparisons between two groups. We detected the differences between the two groups via Student’s *t*-tests. Differences were considered statistically significant at a value of *P* < 0.05. Each experiment was repeated three times.

## 3 Results

### 3.1 Isolation and identification of EXOs

**Figure 1:**
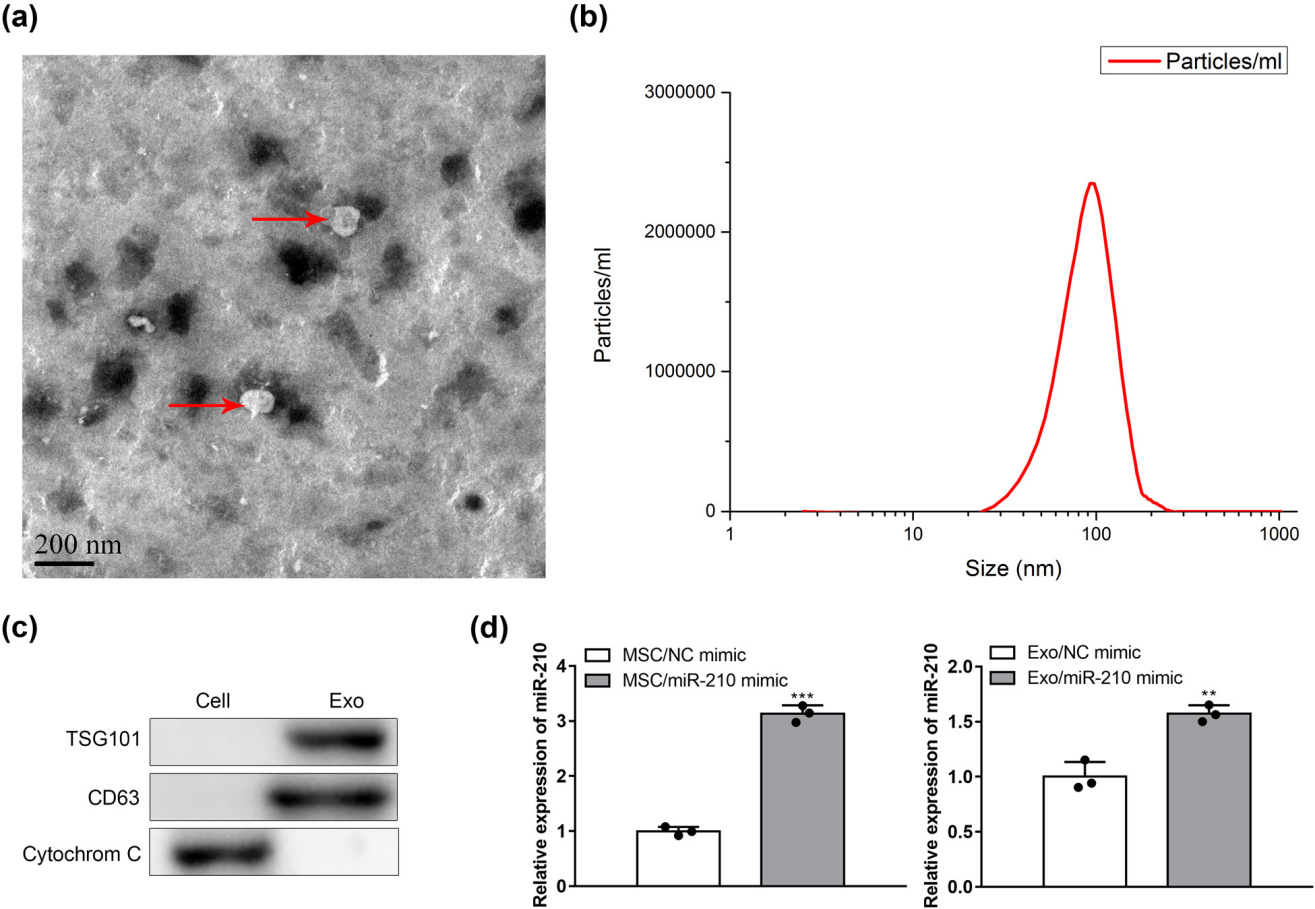
Isolation and identification of MSC-Exos. (a) EXOs morphology revealed by TEM. (b) Particle size distribution measured by nanoparticle tracking analysis. (c) Western blot analysis of EXO surface markers CD63, TSG101, and cytochrome C (*n* = 3). (d) qRT-PCR analysis of miR-210 in MSCs and EXOs: MSCs/NC mimic, MSCs/miR-210 mimic, Exo/NC mimic, Exo/miR-210 mimic (*n* = 3). **P < 0.01, ***P < 0.001 vs MSC/NC mimic.

EXOs were detached from the cultured media of MSCs. First, the EXOs purified were investigated by TEM, nanoparticle tracking analysis, and western blotting. As shown in Figure 1a, typical spherical structures were observed by TEM. Then, nanoparticle tracking analysis showed that these exosomals ranged from 50 and 180 nm in diameter (Figure 1b). Finally, western blotting analysis detected that the EXOs expressed specific surface markers such as TSG101 and CD63, but no expression of the cell-specific marker cytochrome C was detected (Figure 1c).

To detect the microRNA expression of miR-210 in miR-210 transfected MSCs, we used qRT-PCR analysis to determine the miR-210 content in MSCs/NC mimic or MSCs/miR-210 mimic group. Next, we used the qRT-PCR method to detect miR-210 content in the two groups, including Exo/NC mimic and Exo/miR-210 mimic. The qRT-PCR analysis showed that the expression level of miR-210 increased in Exo/miR-210 mimic group compared with Exo/NC mimic group (Figure 1d) (*P* < 0.01)

### 3.2 MSC-Exos localization in microglia

**Figure 2:**
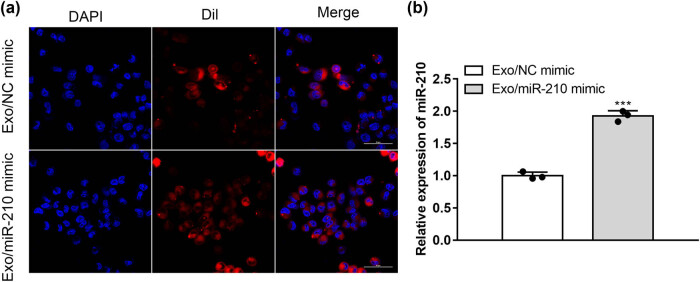
MSC-Exos localization in microglia. Dil-MSC-Exos were co-cultured with microglia for 24 h. (a) Representative immunofluorescence image showing Dil-labeled (red) MSC-Exos inside microglia and with the nuclei stained with DAPI (blue) (*n* = 3). Scale bar: 50 μm. (b) Gene expression of miR-210 as measured by qRT-PCR (*n* = 3). ***P < 0.001 vs Exo/NC mimic.

To determine if microglia can take MSC-Exos, we used PKH26-MSC-Exos to co-culture with target microglia for 24 h *in vitro*. Fluorescence microscopy observed that PKH26-MSC-Exos had been taken up by BV2 microglia, indicating the uptake of PKH26-labeled EXOs into the recipient microglia (Figure 2a).

After that, qRT-PCR analysis was applied to analyze expression level of miR-210. As we expected, the expression level of miR-210 increased in the Exo/miR-210 mimic group compared with Exo/miR-NC mimic group (Figure 2b).

### 3.3 Exosomal miR-210 inhibited the production of NO and pro-inflammatory cytokines

**Figure 3:**
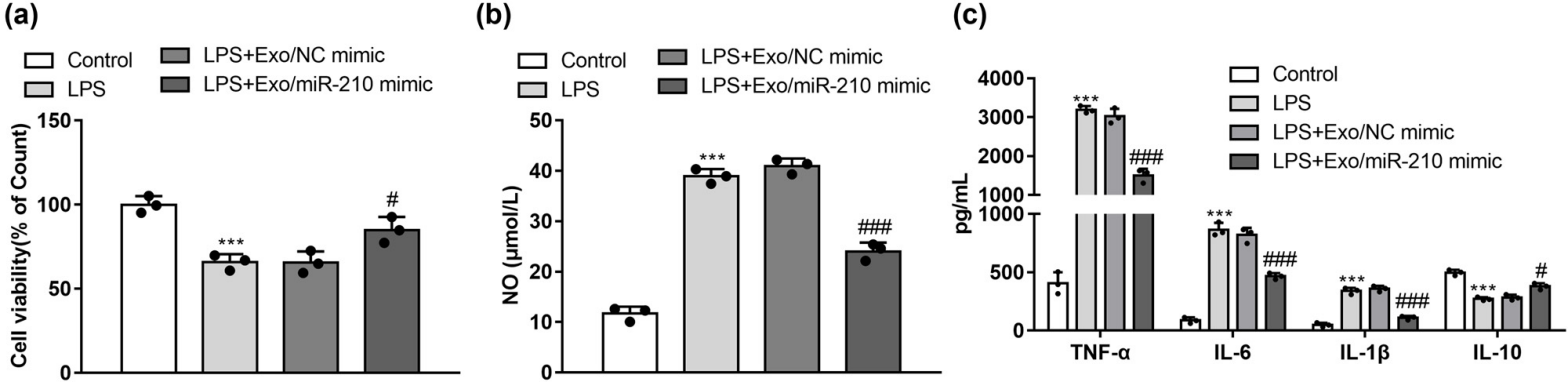
Exosomal miR-210 inhibited LPS-induced NO production and the secretion of pro-inflammatory cytokines. Microglia were stimulated with LPS, followed by Exo/NC mimic or Exo/miR-210 mimic. (a) Viability of microglia assessed using the CCK‐8 method (*n* = 3). (b) NO assay in the supernatant of microglia (*n* = 3). (c) Concentrations of TNF-α, IL-6, IL-1β, and IL-10 were determined by ELISA analysis (*n* = 3). ***P < 0.001 vs Control; *#P* < 0.05, ###*P* < 0.001 vs LPS + Exo/NC mimic.

A CCK‐8 assay detected that LPS treatment down-regulated neuronal cell viability compared to the control group (*P* < 0.05). Compared with the LPS group, Exo/miR-210 mimic treatment promoted neuronal cell viability (*P* < 0.05) (Figure 3a). In addition, LPS treatment promoted M1-related NO production, which plays an essential role in inflammatory regulation [31]. Therefore, we determined the inhibitory effect of Exo/miR-210 mimic on NO concentration in LPS-stimulated microglia. LPS treatment significantly promoted the production of NO in the LPS group (*P* < 0.05), and treatment with Exo/miR-210 mimic significantly reduced the NO production (Figure 3b) (*P* < 0.05).

After that, we evaluated the effects of Exo/miR-210 mimic on the LPS-induced expression of inflammatory factors such as TNF-α, IL-1β, IL-6, and IL-10, which play a vital role in neuroinflammation diseases. The results showed that LPS treatment significantly promoted the expression of TNF-α, IL-6, and IL-1β, while the production of IL-10 decreased in the supernatants of the cells (Figure 3c) (*P* < 0.05). However, Exo/miR-210 mimic were more effective in inhibiting the production of TNF-α, IL-1β, and IL-6 (Figure 3c). Whereas, Exo/miR-210 mimic restores LPS-induced decrease of the production of IL-10 (Figure 3c). In addition, we used an ELISA assay to determine the levels of a pro-inflammatory factor in the microglia supernatant (Figure 3c). The results showed that Exosomal miR-210 significantly reduced the LPS-induced production of pro-inflammatory factors. Exo/miR-210 mimic inhibited the expression of pro-inflammatory cytokines such as IL-6, TNF-α, and IL-1β (*P* < 0.05). The results also showed that Exosomal miR-210 treatment increased the anti-inflammatory cytokines IL-10 compared to the microglia supernatant (*P* < 0.01).

### 3.4 Exosomal miR-210 promoted M1 to M2 phenotypic shift of BV2 microglia cells

Once the stimuli occur in microglia, the cells show two different polarization states after activation. M1 phenotype promote the production of pro-inflammatory cytokines. In addition, the M2 phenotype increases the expression of anti-inflammatory factors [32,33].

**Figure 4:**
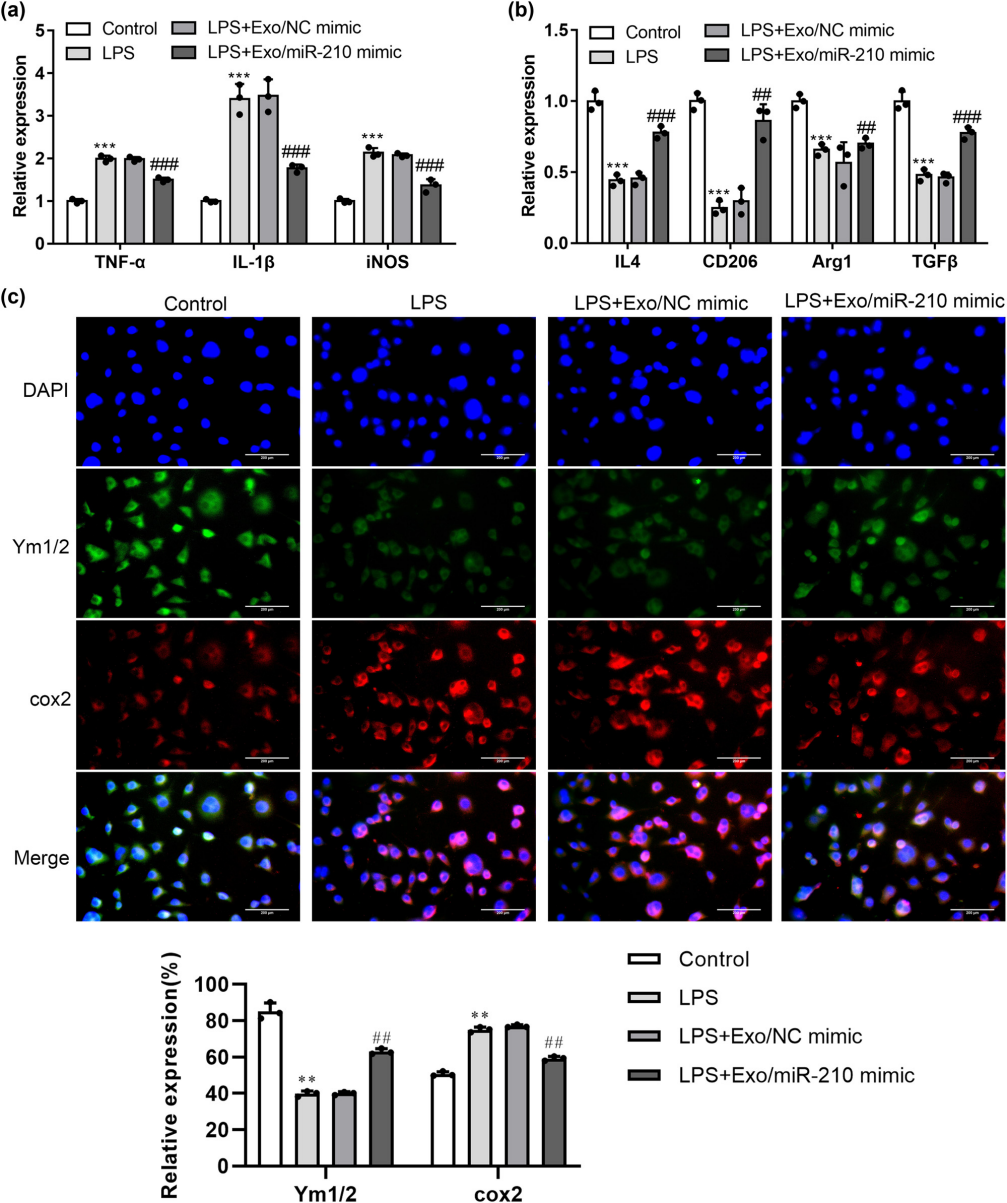
Exosomal miR-210 promoted M1 to M2 phenotypic conversion of microglia. Microglia were stimulated with LPS, followed by treatment with Exo/NC mimic or Exo/miR-210 mimic. (a, b) Expression of M1/M2 markers were determined by qRT-PCR (*n* = 3). (c) Representative images immunostained for M1 microglia and M2 microglia. Cox2 and Ym1/2 expressions per cell were quantified (*n* = 3). **P < 0.05, ***P < 0.001 vs Control; ##*P* < 0.01, ###*P* < 0.001 vs LPS + Exo/NC mimic.

Edaravone plays protective effects on LPS-induced microglia by switching M1/M2 phenotypes and regulating NLRP3 inflammasome activation. TNF-α, iNOS, and IL-1β are specific markers of M1 phenotypes. The specific markers of M2 microglia are IL-4, CD206, Arg1, and TGF-β. To determine the beneficial functions of Exosomal miR-210 on the phenotypic conversion of microglia, we measured TNF-α, iNOS, IL-1β, IL-4, CD206, Arg1, and TGF-β expressions by qRT-PCR. As shown in Figure 4, the mRNA expression of TNF-α, iNOS, and IL-1β increased in activated microglia. Conversely, Exosomal miR-210 could inhibit LPS-induced microglia activation and the mRNA expression of M1-specific membrane markers such as TNF-α, iNOS, and IL-1β decreased (Figure 4a) (*P* < 0.01).

In contrast, the mRNA expression of M2-specific markers such as IL-4, CD206, Arg1, and TGF-β decreased in LPS-induced microglia, while the mRNA expression of M2 phenotype increased in the Exo/miR-210 mimic treatment group (Figure 4b) (*P* < 0.01). Immunofluorescent staining was used to determine the protein expression of M1-associated Cox2 and M2-associated Ym1/2 markers. After Exosomal miR-210 treatment, the number of Cox2 positive microglia decreased, and Ym1/2 positive microglia increased (Figure 4c). In summary, the data showed that Exosomal miR-210 may promote the transition of microglia from a pro-inflammatory M1 phenotype to an anti-inflammatory M2 phenotype *in vitro.*

### 3.5 Exosomal miR-210 enhances the cellular activity of LPS-induced neurons and inhibits apoptosis

**Figure 5:**
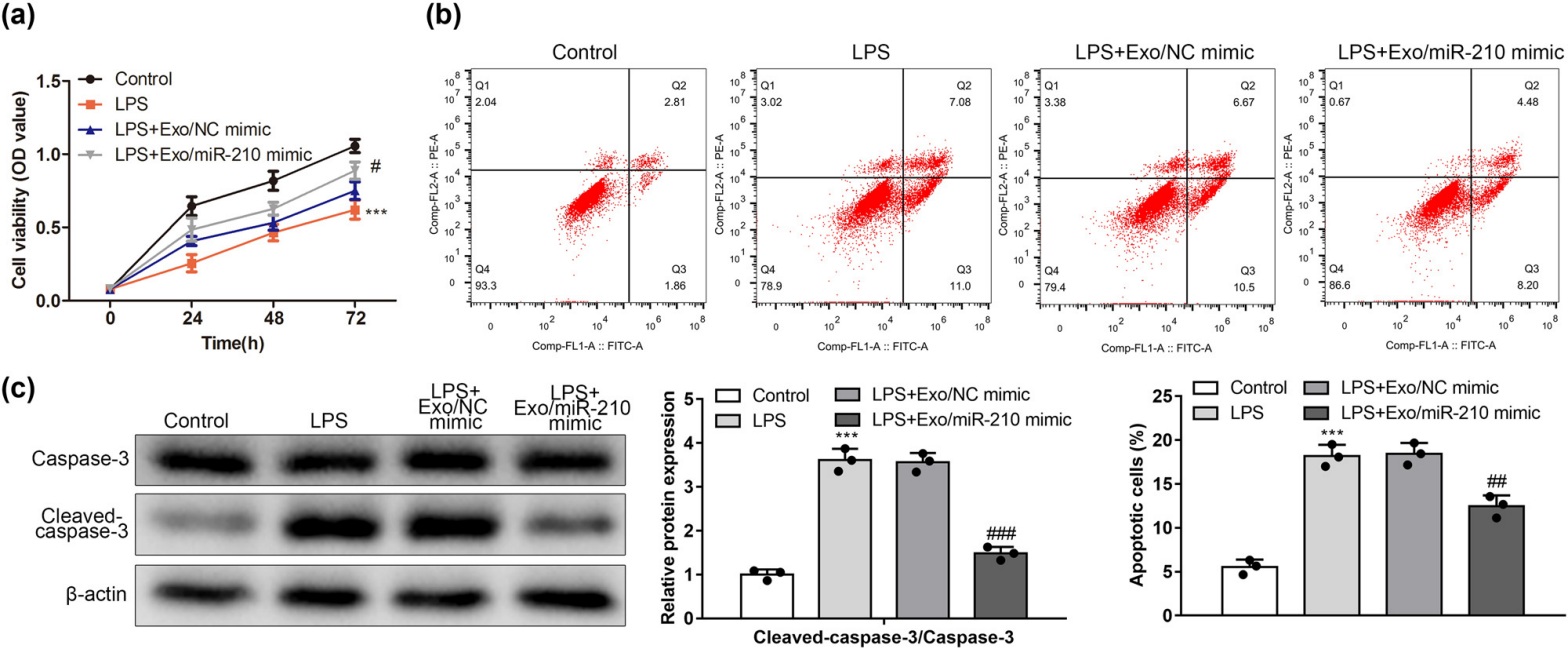
Exosomal miR-210 inhibited microglia-mediated neuroinflammation and reduced neuronal apoptosis. (a) Viability of neuronal HT22 cells was assessed using the CCK‐8 method. (b) Percentage of apoptotic neuronal HT22 cells was assessed using flow cytometry (*n* = 3). (c) Representative immunoblots were probed with antibodies against Cleaved‐caspase-3, caspase-3, and β‐actin (*n* = 3). Quantification of the level of Cleaved‐caspase-3 normalized to β‐actin. Quantification of the level of caspase-3 normalized to β‐actin. ***P < 0.001 vs Control; *#P* < 0.05, ##*P* < 0.01, ###*P* < 0.001 vs LPS + Exo/NC mimic.

Neuronal inflammation plays a vital role in neuronal cell dysfunction and apoptosis. A CCK‐8 assay revealed that Exosomal miR-210 promoted the viability of neuronal HT22 cells when compared to cells in the LPS + Exo/NC mimic group (Figure 5a) (*P* < 0.05).

To detect the effects of Exosomal miR-210 on LPS-stimulated neuronal apoptosis, we used flow cytometry to identify apoptotic neurons. After Exo/miR-210 mimic treatment, the number of apoptotic cells decreased compared with the LPS + Exo/NC mimic group (Figure 5b) (*P* < 0.05). Furthermore, as shown in Figure 5c, western blotting showed that LPS promoted proapoptotic proteins, including cleaved caspase-3 and caspase-3, and the treatment of Exosomal miR-210 inhibited the protein expression of cleaved caspase-3 and caspase-3 (Figure 5c) (*P* < 0.05). Taken together, these data indicated that Exosomal miR-210 may be involved in inhibiting the development of neuroinflammatory response.

### 3.6 Exosomal miR-210 promoted neurogenesis

**Figure 6:**
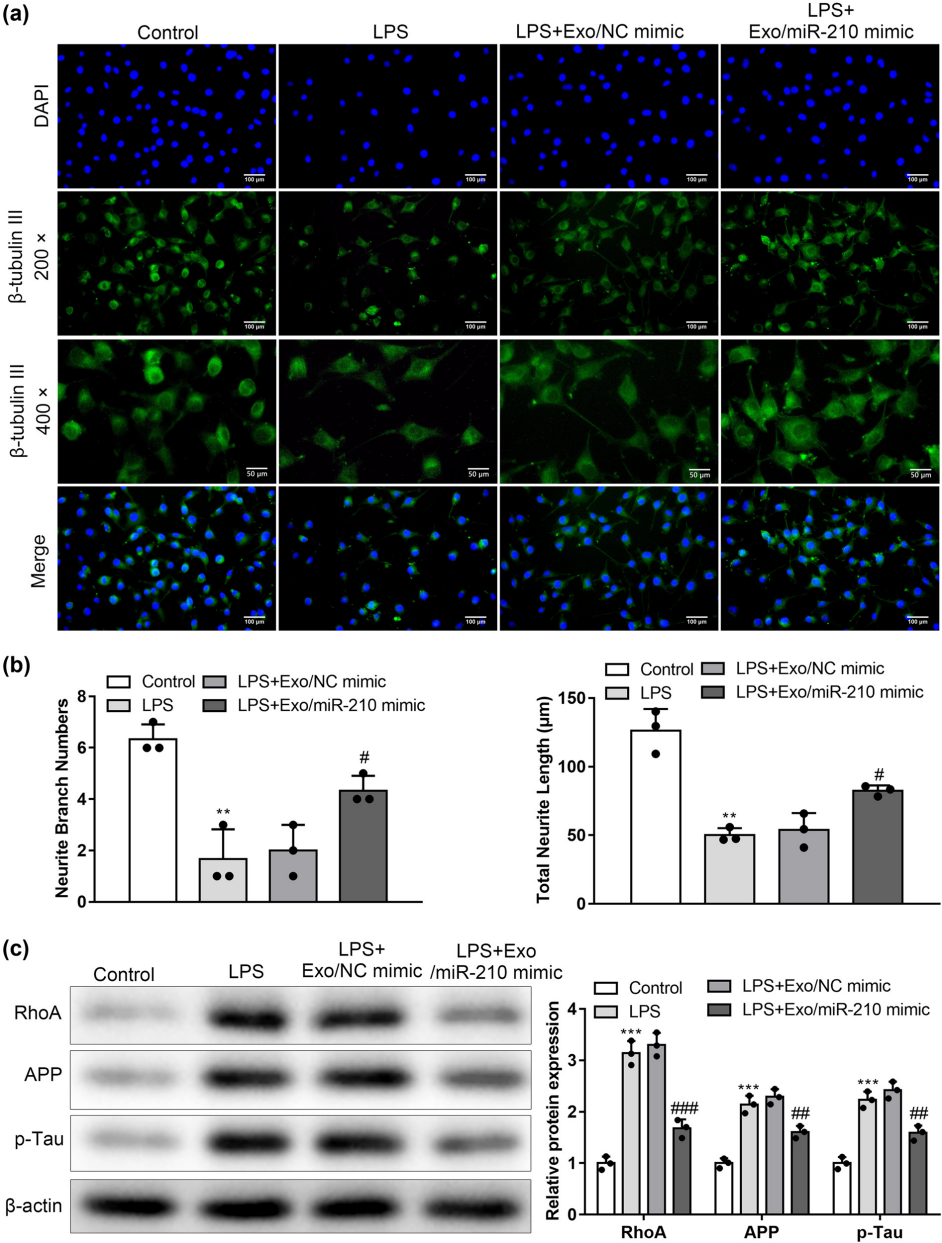
Exosomal miR-210 promoted neurogenesis. (a) Representative images of cultured neuronal cells immunostained with anti-β-tubulin III antibody (*n* = 3). Scale bars: 50 and 100 μm. (b) Effect of Exosomal miR-210 on the axonal length and neurite branching in neuronal cells. Axonal length in different groups measured via imageJ. The average of three counts was calculated for the axonal length, and all the samples were analyzed over 30 cells per experiment (*n* = 3). (c) Detection of RhoA, APP, and p-Tau in the brain by western blot (*n* = 3). **P < 0.01, ***P < 0.001 vs Control; #*P* < 0.05, ##*P* < 0.01, ###*P* < 0.001 vs LPS + Exo/NC mimic.

To detect the effects of Exosomal miR-210 on neurogenesis, we observed the therapeutic effect on neuronal cells. Compared with the control group, LPS treatment decreased β3-tubulin favorable neurite elongation. In addition, the treatment of Exosomal miR-210 extraordinarily increased the length of the β3-tubulin-positive neuronal cell (Figure 6a) (*P* < 0.05). These neurons were stained with a β3-tubulin antibody. The number of neurite branches is defined as the number of neurites on the neurons’ somatic cells. Select three neurons with the largest number of neurites in each group of visual fields, count their number of neurite branches, and calculate the average value. In addition, the length of the neurite is defined as the length from the somatic cell of the neuron to the end of the neurite. Three neurons with the longest neurites in each group were selected, and the length of neurites was measured using the ruler function of imageJ. The treatment of LPS significantly decreased the length of neuronal cells and the number of branches (*P* < 0.05). Exosomal miR-210 treatment rescued the reduction in the neurite length and neurite branching (Figure 6b) (*P* < 0.05). A recent study determined that the p-Tau protein played a vital role in neuronal apoptosis. Aberrant expression and denaturation of Tau and APP can lead to neuronal cell death [34]. Compared with the control group, LPS increased RhoA, APP, and p-Tau protein. To evaluate the Exosomal miR-210 effect on the formation of RhoA, APP, and p-Tau, western blots confirmed that Exosomal miR-210 decreased RhoA, APP, and p-tau protein levels (Figure 6c).

## 4 Discussion

This study showed that EXOs from MSCs overexpressing miR-210 play an essential role in inhibiting neuroinflammation. In addition, we analyzed the mechanism of the anti-inflammatory effect of neuroinflammation. The main findings from our study are listed as follows: (1) Exosomal miR-210 inhibited LPS-induced production of NO and pro-inflammatory factors. (2) Exosomal miR-210 derived from MSCs could promote M1/M2 phenotype conversion and transform the pro-inflammatory M1 microglia into beneficial anti-inflammatory M2 microglia. (3) Exosomal miR-210 suppressed microglia-mediated neuroinflammation and reduced neuronal apoptosis. (4) Exosomal miR-210 increased the neurite length and the number of branches. Thus, exosomal miR-210 derived from MSCs promoted neurogenesis.

Our findings reported that Exosomal miR-210 inhibited neuronal inflammation and contributed to neurogenesis through inhibiting microglia M1/M2 phenotype conversion-mediated inflammatory response. In a recent study, MSC-Exos have been determined as an essential factor in the development of neuronal inflammation [23,35]. Furthermore, in the traumatic brain injury model, it has been confirmed that MSCs-derived EXOs significantly promoted neuronal recovery by inhibiting the development of neuroinflammation [36]. They are certain paracrine factors in a nanoscale size and great content (lipids, proteins, mRNA, and miRNAs), enabling them to mediate information exchange between cells and tissues at close and long distances. EXOs are one of the particular paracrine factors in a nanometer size (50–150 nm). Their great contents, including lipids, proteins, and miRNAs, transform the information between cells and tissues. In addition, the EXOs protect during transportation. The recent studies confirmed that microglia could efficiently take in fluorescence-labeled MSCs-Exos *in vitro* [37]. Furthermore, microglia could take up EXOs by various endocytic pathways, including macropinocytosis and caveolin-mediated uptake [38], even by plasma membrane fusion [39].

As an essential component in MSC-Exos, miRNAs have attracted the attention of researchers [40]. miRNA is a kind of small RNA that combines with target RNA to silence the genes. Therefore, miRNAs may participate in regulating complex signaling networks and have therapeutic potential in neuronal disease. In addition, several miRNAs participate in modulating microglia polarization and regulating M1/M2 phenotype conversion. Zaccagnini et al. found that miR-210 overexpression inhibited inflammation and promoted muscle damage recovery *in vivo* [41]. In murine macrophages, the treatment of LPS promotes the expression of miR-210, which reduces the secretion of pro-inflammatory cytokine [27]. In the articular cartilage of osteoarthritis rats, miR-210 could inhibit the production of pro-inflammatory factors by regulating the NF-κB signaling pathway.

In summary, these findings suggested that miR-210 could inhibit inflammation. However, MiR-210 has been shown to promote the development of inflammation in acute colitis [42]. In addition, miR-210 inhibits the STAT6/IL-4 anti-inflammatory pathway in cytotrophoblasts and promotes maternal inflammation activation [43]. Thus, in different inflammatory diseases, the regulatory effects of miR-210 may be different.

Generally, we found that the anti-inflammatory properties of Exosomal miR-210 were associated with modulating microglia polarization. Exosomal miR-210 ameliorated the secretion of pro-inflammatory factors and promoted anti-inflammatory factors in LPS-induced BV2 microglia cells. Immunofluorescence staining and the qRT-PCR method showed that Exosomal miR-210 promoted pro-inflammatory M1 phenotype conversion to anti-inflammatory M2 phenotype. Therefore, Exosomal miR-210 may inhibit neuronal inflammation effects through modulating microglia polarization. We determined that miR-210 could reduce microglia-associated inflammation and also extraordinarily protect against the apoptosis of neural cells.

In summary, miR-210 inhibited neuronal inflammation and promoted neurogenesis. However, it modulates these processes through many other mechanisms that are yet to be identified. Therefore, we will explore other mechanisms that may participate in the future regulatory network of microglia polarization and inflammation.

All in all, our findings demonstrate that EXOs derived from MSCs overexpressing miR-210 reduced microglia-associated inflammation and promoted neurogenesis. Therefore, our study provides new insight into a potential therapeutic target of miR-210 in the modulation of microglia-mediated neuroinflammation, which may be beneficial for treating cerebrovascular and neurodegenerative disorders.

However, our study still has some limitations. For example, whether miR-210 mimic can affect the inflammatory level of nerve cells through the polarization of microglia has not been verified at the *in vivo* level. In addition, miR-210 influences neuronal or microglial polarization by regulating downstream target genes, which still needs further study.

## 5 Conclusion

Our study found that Exosomal miR-210 inhibited neuroinflammation and contributes to neurite outgrowth possibly by modulating microglia polarization.

## Appendix

**Table A1:** Primers used in RT-PCR experiments

Primer	Forward (5′–3′)	Reverse (5′–3′)
Tgf-β	GGCACCATCCATGACATGAACCG	GCCGTACACAGCAGTTCTTCTCTG
Arg1	GTCTGAACAAGCACTGTGAAAG	GGAAGCCAA ATACGACACTAAG
CD206	CCACTCTATCCACCTTCAC	GCCTCAATCCAACCAAAC
Il-4	GTAAACGAGGCTTCCTGTC	CCCAGAATCCAGTCTTTCC
iNOS	TCCTACACCACACCAAAC	CTCCAATCTCTGCCTATCC
Il-1β	ATCTCACAGCAGCATCTCGACAAG	CACACTAGCAGGTCGTCATCATCC
Tnf-α	GCATGATCCGAGATGTGGAACTGG	CGCCACGAGCAGGAATGAGAAG
Gapdh	ATGGGGAAGGTGAAGGTCG	GGGGTCATTGATGGCAATA
miR-210	CGCCTGTGCGTGTGACAGCG	GTGCAGGGTCCGAGGT
U6	CTCGCTTCGGCAGCACATATACTA	ACGAATTTGCGTGTCATCCTTGC
